# *Ex Vivo* Pathomechanics of the Canine Pond-Nuki Model

**DOI:** 10.1371/journal.pone.0081383

**Published:** 2013-12-13

**Authors:** Antonio Pozzi, Stanley E. Kim, Bryan P. Conrad, MaryBeth Horodyski, Scott A. Banks

**Affiliations:** 1 Comparative Orthopaedics Biomechanics Laboratory, Department of Small Animal Clinical Sciences, University of Florida, Gainesville, Florida, United States of America; 2 Comparative Orthopaedics Biomechanics Laboratory, Department of Orthopaedics and Rehabilitation, University of Florida, Gainesville, Florida, United States of America; 3 Comparative Orthopaedics Biomechanics Laboratory, Department of Mechanical and Aerospace Engineering, University of Florida, Gainesville, Florida, United States of America; University of Sydney, Australia

## Abstract

**Background:**

Transection of the canine cranial cruciate ligament (CCL) is a well-established osteoarthritis (OA) model. The effect of CCL loss on contact pressure and joint alignment has not been quantified for stifle loading in standing. The purposes of the study were to measure femorotibial contact areas and stresses and joint alignment following transection of the CCL in an ex vivo model. We hypothesized that transection of the CCL would lead to abnormal kinematics, as well as alterations in contact mechanics of the femorotibial joint.

**Methodology/Principal Findings:**

Eight canine hindlimbs were tested in a servo-hydraulic materials testing machine using a custom made femoral jig. Contact area and pressure measurements, and femorotibial rotations and translations were measured in the normal and the CCL–deficient stifle in both standing and deep flexion angles.

We found that at standing angle, transection of the CCL caused cranial translation and internal rotation of the tibia with a concurrent caudal shift of the contact area, an increase in peak pressure and a decrease in contact area. These changes were not noted in deep flexion. At standing, loss of CCL caused a redistribution of the joint pressure, with the caudal region of the compartment being overloaded and the rest of the joint being underloaded.

**Conclusion:**

In the Pond-Nuki model alterations in joint alignment are correlated with shifting of the contact points to infrequently loaded areas of the tibial plateau. The results of this study suggest that this cadaveric Pond-Nuki model simulates the biomechanical changes previously reported in the in-vivo Pond-Nuki model.

## Introduction

Canine unilateral cranial cruciate ligament (CCL) transection has been the most commonly used model for producing osteoarthritis (OA) since it was first described by Pond and Nuki in the early 1970s [1]. Varying degrees of cartilage changes, osteophyte formation and meniscal fibrillation occur following CCL transection [2], [3], [4], [5]. An in vivo, long-term study evaluating three-dimensional kinematics in dogs reported a consistent pattern of cranial tibial translation and frontal-plane instability immediately after CCL transection, which did not improve with time [6]. Without the stability afforded by the CCL, the femoral condyles slide down the caudally sloped tibial plateau resulting in cranial displacement of the tibia relative to the femur [7]. Abnormal dynamic joint function subsequent to loss of the integrity of the CCL is purported to contribute to the development of OA by influencing the mechanobiology of articular cartilage, though the exact relationship has not been definitely elucidated [8]. A recent study in CCL-deficient dogs showed that abnormal articular surface interactions may be a mechanism that initiates OA development [9].

The causes of articular cartilage degeneration are complex and involve interrelated biological, mechanical, and structural pathways [10], [11], [12]. The ex vivo pathomechanics of OA has been described by Andriacchi as a framework divided in an initiation and a progression phase [13]. The initiation phase is characterized by kinematic changes associated with a shift in load-bearing regions, while the progression phase occurs as the disease progresses more rapidly with increased loads [13], [14]. Cartilage metabolism is dependent on the maintenance of the mechanical stimuli for which the chondrocytes are adapted [11], [15]. Therefore OA may be triggered by reduced loading, which activates the subchondral growth front by reducing fluid pressure, or by increased loading causing mechanical damage at the articulating surfaces [13]. An understanding of how the joint kinematics and contact mechanics of the stifle are altered by CCL transection may be important in order to relate the aberrant biomechanics to the process of degeneration observed in the Pond-Nuki model [1]. If mechanical factors associated with OA can be identified soon after joint instability develops, perhaps treatment strategies can be developed to stop the progression of OA in the early phase of the disease.

The effect of conformational altering tibial osteotomy on contact mechanics and joint alignment have been recently studied in a canine cadaveric model [16]. While preliminary data on the effect of CCL transection were collected in that study, a sham osteotomy was performed before collecting data. It would be important to specifically measure the effect of CCL transection on the canine stifle, without interaction of other treatments. The purposes of the study were to evaluate the effects of CCL transection on femorotibial contact areas and stresses and joint alignment in the stifle. We hypothesized that transection of the CCL would lead to alterations in contact mechanics of the femorotibial joint secondary to cranial subluxation and internal rotation of the tibia.

## Materials and Methods

### Ethics Statement

This study was carried out in strict accordance with the recommendations in the Guide for the Care and Use of Laboratory Animals of the National Institutes of Health. All procedures in the study were approved by the Institutional Animal Care and Use Committee of University of Florida (IACUC Number: E810). The hind limbs used in this study were obtained from dogs which were euthanized under a different project (IACUC Number: 200902382). The PI of this study obtained permission to use dogs euthanized at the local shelter. Euthanasia was humanely performed using pentobarbital and phenytoin solution.

### Specimen Preparation

Eight hind limbs (four pairs) were harvested by disarticulation of the coxofemoral joint from four adult dogs weighing between 28 to 35 kg that were euthanatized for reasons unrelated to the study. Frontal and sagittal view radiographs were taken of each limb to ensure there was no radiographic evidence of stifle pathology. The tibial plateau angle was measured for each limb on the sagittal view radiographs, using previously reported methods [17]. After imaging, all musculature was dissected from the limbs while carefully preserving the stifle and hock joint capsules, collateral ligaments, and all soft tissue distal to the hock joint. The specimens were wrapped in saline-soaked towels and stored at −20° Celsius until testing.

**Figure 1:**
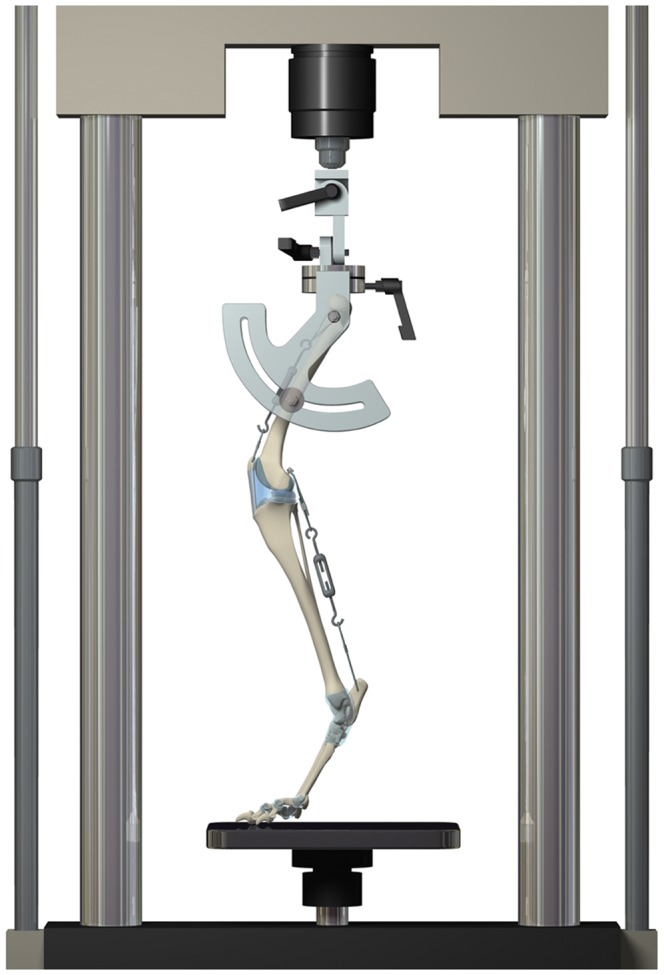
Schematic illustration of the prepared specimen mounted to a materials testing machine. Stifle and hock angles of 135±5° were attained with the limb subjected to an axial load of 30% body weight.

In preparation for testing, the limbs were thawed to room temperature. Tissues were kept moist throughout the experiment by spraying the specimens with isotonic saline. In each specimen, braided steel cable was passed through a 2.5 mm diameter hole drilled transversely through the widest portion of the patella and secured into a small loop. A turnbuckle links and braided steel cables were used to mimic the quadriceps mechanism and the gastrocnemius muscle. Three nylon screws (McMaster-Carr Supply company, Cleveland, OH) were implanted into the femur and tibia as landmarks for determining the three-dimensional, static pose of the stifle during testing. The specimen to be tested was linked to a custom femoral jig with two 4 mm threaded rods placed in a lateral-to-medial direction at the neck and the mid-diaphysis of the femur. The femoral jig, which mounted directly to a servo-hydraulic materials testing machine, was designed to permit adjustment of ‘hip’ flexion, adduction/abduction and axial rotation angle (Figure 1). During loading, flexion and adduction/abduction hinges on the femoral jig were constrained, while axial rotation was left unconstrained.

Instantaneous intra-articular contact area and pressure measurements were obtained using the I-Scan system (Tekscan Inc., Sounth Boston, MS), consisting of a custom designed, plastic laminated, thin-film (0.1 mm) electronic pressure sensor, sensor handle, and Tekscan software. The sensors had two sensing areas of 30.9 mm×12.0 mm, a pressure sensitivity of 0.01 MPa, and a pressure range of 0.5 to 30.0 MPa. Each new sensor was conditioned and calibrated according to the manufacturer's guidelines immediately prior to testing of each specimen. Following calibration, the sensor was placed subjacent to the menisci by creating cranial and caudal horizontal capsulotomies in the medial and lateral stifle compartments and in place by gluing and suturing the peripheral tabs to Kirschner wires implanted at the cranial aspect of the tibial plateau.

### Testing Protocol

With the specimen mounted to the Instron in an unloaded state, the locations of the cranial and caudal margins of the medial and lateral tibial condyles on the contact maps were identified with the I-scan software by applying gentle pressure to the overlying sensing elements with a probe. The turnbuckles could be adjusted to attain a stifle and ankle angle of 135±5° (stance phase angle), corresponding to the mid-point of stance phase of gait during walking, or 90±5° (high-flexion angle) corresponding to a flexed position of the hind limb. The joint angles were measured with a plastic goniometer during loading, with the arms of the goniometer aligned to the tibial and femoral diaphyses. The paw of the specimen was in contact with, but was not fixed to, the Instron actuator table during loading. To reproduce standing, a static axial load of 30% body weight was applied by the Instron. Prior to data acquisition, the limb was initially loaded with the adduction/abduction hinge of the femoral jig unconstrained. By monitoring the real-time output of the I-scan system, the adduction/abduction hinge was then locked in a position that resulted in a 50∶50±10% medial-to-lateral force distribution across the normal stifle.

Loading of each specimen was performed before and after transection of the CCL at its origin via a caudal approach to the stifle. Turnbuckles were adjusted throughout the experiment to maintain stifle and ankle angles of either 135±5° or 90±5°. Loading and data acquisition was performed in the sequence: 1) CCL intact/high flexion; 2) CCL intact/stance phase angle; 3) CCL deficient/stance phase angle; 4) CCL deficient/high-flexion angle. For each condition, the contact area and pressure measurements were acquired after maintaining peak force for 5 seconds. While the specimen was loaded, the static, three-dimensional pose of the tibial and femoral nylon screws were digitized using a Microscribe 3DX digitizing arm (Immersion Corp., San Jose, CA), which possesses an accuracy of 0.23 mm.

### Data Analysis

**Figure 2:**
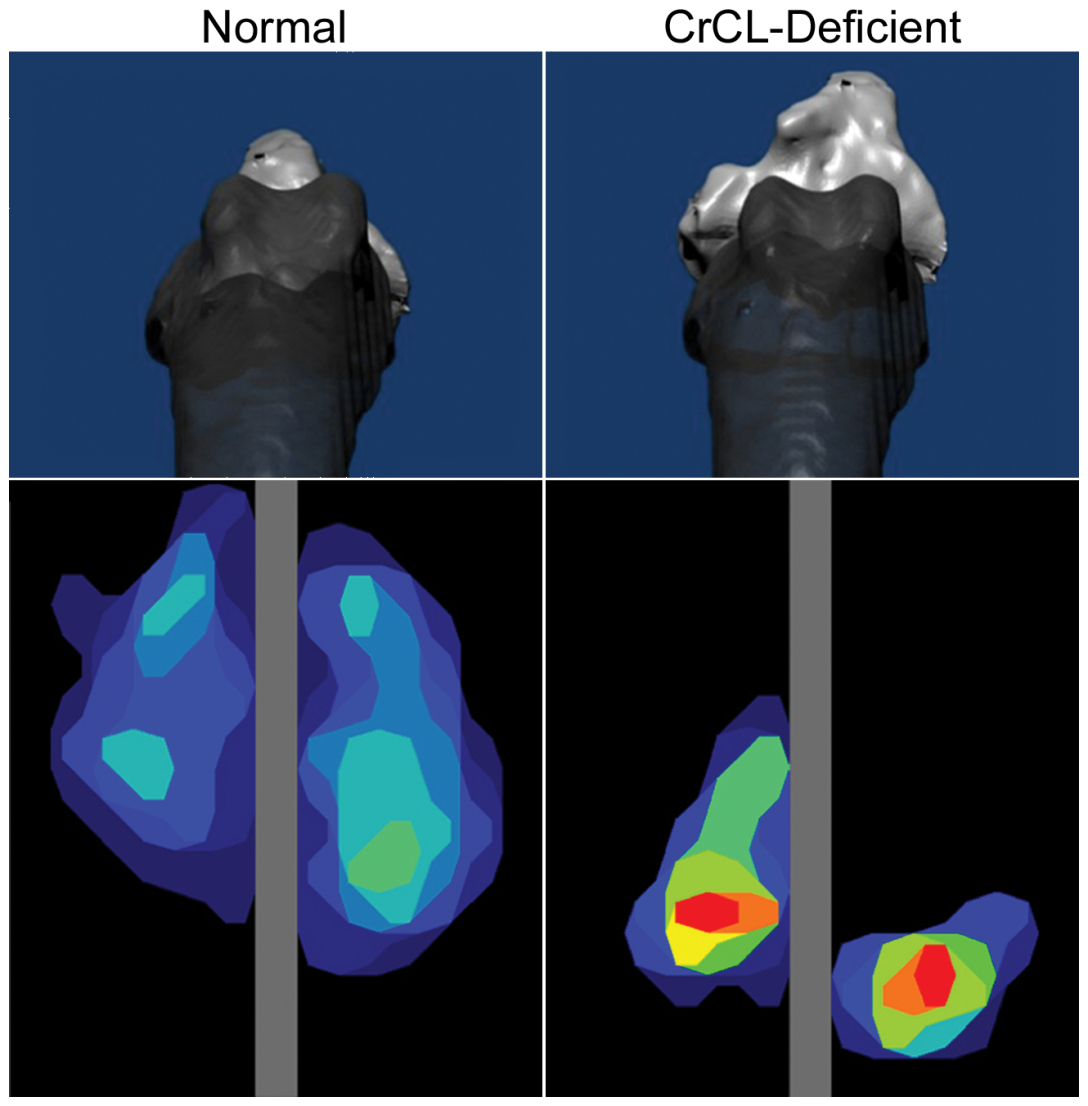
Axial view of bone models depicting three-dimensional poses of normal and cranial cruciate ligament (CCL)-deficient stifles in weight bearing position, with corresponding contact maps representative of each testing condition. The tibia (light gray) is cranially displaced and internally rotated relative to the femur (dark gray) after CCL transection. CCL transection resulted in caudal shift, reduced area and increased pressure of femorotibial contact; Left = lateral, top = cranial.

The I-scan software was used to generate a contact map and measure the contact area, mean contact pressure and peak contact pressure in the combined (medial+lateral), medial and lateral stifle compartments (Figure 2). Contact area was defined as the area of contact between the tibial plateau, the femoral condyle, and the portion of the meniscus loaded by the femur. Peak contact pressure was defined as the highest pressure measured in the contact area, whereas mean contact pressure represented the average of the pressures across the contact area. Pressure distribution was described according to the location of the peak pressure in each stifle compartment: the relative location of peak pressure for each condition was defined as the distance from the peak pressure sensel to the caudal margin of the tibial condyle (medial or lateral) in the sagittal plane, divided by the entire length of the tibial condyle in the sagittal plane (Figure 2). Pressure distribution was further characterized by dividing each stifle compartment into three regions of equal size (cranial, central, caudal) and measuring the absolute contact force in each region.

Following testing, CT images of the femurs and tibiae, with nylon screws in place, were acquired. Bone segmentation was performed on the Slicomatic software package (Tomovision, Montreal, QC, Canada), and three-dimensional bone models for the tibiae and femurs of each specimen were created using Geomagic software (Goemagic Inc., Research Triangle Park, NC). Locations of anatomical landmarks for the femur (center of the lateral and medial condyles, center of the femoral head, and origin of CCL) and the tibia (outermost edge of the lateral and medial condyles, center of the distal end of the tibia, insertion of CCL), and femoral and tibial nylon screw locations were identified in the 3D bone models. Rotations of the tibia relative to the femur were calculated using body-fixed axes in the order (flexion/extension, adduction/abduction, internal/external rotation), corresponding to the rotational component of the joint coordinate system described by Grood and Suntay [18]. Translations of the tibia relative to the femur were measured from CCL origin to insertion, and expressed in an orthogonal anatomic coordinate system fixed to the tibia [6]. Calculations were performed on a custom written computer program using Matlab (The MathWorks Inc., Natick, MA).

### Statistical Analysis

Paired t-tests were used to evaluate differences in contact area, mean contact pressure, peak contact pressure, and pressure distribution in the combined and individual (medial and lateral) stifle compartments, between the normal and the CCL –deficient stifle in both 135±5° and 90±5° flexion angles. Paired t-tests were also used to evaluate differences in internal-external tibial rotation, cranial-caudal tibial translation, proximal-distal tibial translation, and stifle flexion angle between the normal and the CCL –deficient stifle in both 135±5° and 90±5° flexion angles. Contact force of each region (cranial, middle, caudal) of each compartment was compared with a general linear model. For all statistical analyses performed, P<0.01 was considered statistically significant. A statistical analysis software package (SPSS 16.0, SPSS Inc., Chicago, IL) was used to perform all statistical analyses.

## Results

Mean body weight of the dogs was 32±3 kg. The mean caudal tibial plateau angle measured 23°±1°. As determined by the analysis of the joint alignment, stifle angles slightly varied between the normal (138±3.5°) and CCL-deficient (142.7±2.8°) conditions at the stance phase angle (P = 0.007), but not at the high-flexed angle (P = 0.572).

Mean values for the contact mechanics are provided in table 1 and 2 and joint alignment parameters are provided in Table 3. In the normal condition with the stifle at a stance phase angle CCL transection caused a significant cranial translation and internal rotation of the tibia (P<0.001). At the high-flexion angle, no significant changes in alignment were noted between normal and CCL-deficient stifles. At this angle, transecting the CCL did not cause any significant translations or rotations from the original position of the joint.

**Table 1:** Contact mechanics data for the normal and CCL-deficient stifles positioned at 135° and 90° flexion.

Flexion angle (degrees)		135		90	
CCL status		Intact	Transected	Intact	Transected
	Total	316±34	177±27*	316±31	291±32
**Contact area (mm^2^)**	Medial	177±19	73±13*	174±22	155±16
	Lateral	138±26	104±24	142±20	135±30
**Peak Contact Pressure (MPa)**	Medial	3.1±0.6	5.6±1.2*	4.0±0.8	4.1±0.9
	Lateral	3.0±0.5	4.1±1.4	4.0±0.4	3.5±0.8
	Total	1.4±0.2	1.5±0.2	1.5±0.2	1.5±0.3
**Mean Contact Pressure (MPa)**	Medial	1.4±0.2	1.9±0.4	1.5±0.3	1.6±0.2
	Lateral	1.3±0.3	1.3±0.2	1.5±0.3	1.4±0.3
**Peak Pressure Location (%)**	Medial	50±15	16±3*	39±7	40±8
	Lateral	61±9	23±9*	49±5	52±10

*) indicate significant differences (P<0.01) between intact and transected conditions. Peak pressure location was defined as the distance from the peak pressure sensel to the caudal margin of the tibial condyle (medial or lateral) in the sagittal plane, divided by the entire length of the tibial condyle in the sagittal plane. Asterisks (

**Table 2:** Contact force distribution for the normal and CCL-deficient stifles positioned at 135° and 90° flexion angles.

Flexion angle (degrees)		135		90	
CCL status		Intact	Transected	Intact	Transected
**Medial compartment contact force (N)**	**Cranial Central Caudal**	72±28	0±0*	34±14	30±21
		130±22	2±2*	143±50	140±37
		45±30	135±30*	93±51	86±65
**Lateral compartment contact force (N)**	**Cranial Central Caudal**	75±18	2±3*	40±20	33±15
		108±26	46±13*	156±43	136±33
		1±1	94±20*	6±9	9±13

*) indicate significant differences (P<0.01) between intact and transected conditions. Asterisks (

**Table 3:** Static three-dimensional femorotibial poses for normal and cranial cruciate ligament (CCL)-deficient stifles positioned at 135° and 90° flexion angles.

Flexion angle (degrees)		135		90	
CCL status		Intact	Transected	Intact	Transected
**Translations (mm)**	**Ca-Cr**	10.2±3.9	25.1±4.9*	10.4±3.6	11.0±4.0
	**M-L**	9.1±5.2	10.6±4.7	−0.7±10.8	−1.1±10.8
	**D-P**	4.6±2.2	−2.5±2.5	3.8±3.6	3.6±3.1
**Rotations (degrees)**	**F-E**	138.5±3.5	142.7±2.8*	101.5±5.5	102.7±7.6
	**E-I**	13.8±3.6	−4.6±5.7*	10.7±3.6	−8.3±4.2
	**Vr-Vl**	10.4±2.8	5.3±2.3	2.8±7.4	2.4±7.3

*) indicate significant differences (P<0.01) between intact and transected conditions. For the translational variables, positive values indicate cranial, distracted and medial positions of the tibia relative to the femur. For the rotational variables, positive values indicate greater stifle extension, external tibial rotation, and varus. Asterisks (

Mean values for the contact mechanics parameters are provided in Table 2. At the stance phase angle, CCL transection resulted in significant changes to all parameters for the combined and medial stifle compartments, while significant differences were less apparent in the lateral compartment. CCL transection resulted in significant decrease in total and medial contact area and significant increase in total and medial compartmental peak pressure (P<0.001).

At the stance phase angle, the medial peak pressure was located at 50±15% of the medial tibial condyle length from the caudal margin of the medial compartment in stifles prior to CCL transection at the stance phase angle. Peak pressure in the medial compartment shifted caudally to 16±3% after CCL transection (P<0.001). There was also a significant caudal shift in peak pressure location in the lateral compartment after CCL transection, from 61±9% of the lateral tibial condyle length from the caudal margin of the lateral tibial condyle, to 23±9% (P<0.001). No significant changes in any contact mechanics outcome measure were noted after CCL transection at the high-flexion angle.

## Discussion

Our study supports the hypotheses that changes in stifle alignment alter contact areas and stresses following CCL transection in dogs. The reported kinematics data are consistent with previously reported in vivo studies on the Pond-Nuki model [6], [19], which described cranial-caudal and rotational instability following transection of the CCL. However, the advantage of our experimental design was that joint alignment and contact areas and stresses were simultaneously measured.

Compared to in vivo studies [6], [19], some differences were noted in the magnitude of translations and rotation following CCL transection. For instance, we noted a mean of 15.2 mm cranial tibial translation after CCL transection, approximately 50% greater than the equivalent in vivo results obtained by Korvick et al [19]. The greater tibial translation and rotation noted in our model may be caused by the dissection of the joint capsule and the menisci, as well as the lack of balanced muscle forces, which are important stifle stabilizers after transection of the CCL. Furthermore, dogs analyzed with in vivo kinematics may have compensated for loss of the CCL by reducing the external load on the limb and carrying the limb in greater flexion [6]. In our model the axial load was not decreased after transection of the CCL to allow for comparison of cartilage stress between conditions. A decrease in axial load as expected in a dog with a CCL deficient stifle may have resulted in decreased tibial translation and rotation.

Malalignment of the joint caused a caudal shift of the contact area, an increase in peak pressure and a decrease in contact area. The positional offset shifted the load-bearing contact areas of the tibial plateau away from the normal locations. Previous researchers have reported that loading regions of articular cartilage not accustomed to load may lead to OA, which can progress more quickly with increased stresses on the cartilage [20], [21]. In our model the medial compartment experienced greater changes in contact mechanics which may explain the severe cartilage degeneration observed in the caudomedial and posterior medial compartments of chronic cruciate-deficient stifles in dogs and human patients, respectively [22]. This lesion, called “cupula” in the French literature, differs from the typical anteromedial wear pattern observed when the anterior cruciate ligament (ACL) is intact and has been ascribed to a posterior shift and increase in contact pressure [22], [23]. Based on our results, the mechanism of the Pond-Nuki model is consistent with both theoretical models [24] and human in vivo studies showing changes in articular surface contact location in unstable joints [25], [26]. Our results confirm that the theory of pathomechanics of OA can be applied to the Pond-Nuki model [13].

We found more severe changes in joint alignment and contact mechanics in stifles positioned in the extension than stifles in the flexion position. The CCL-deficient joint at 90° flexion did not subluxate and had only mild changes in contact mechanics. This is consistent with the alignment data reported by Griffith on the human ACL-deficient knee with increased posterior tibial slope [27]. In the human knee, the anterior shift of the tibia was highest with the knee extended and decreased with flexion [27]. The effect of knee flexion angle on joint stability may be explained by considering both the orientation of the posterior tibial slope and the muscular forces. It has been suggested that the most significant compressive force acting at the knee joint may be oriented parallel to the patellar tendon or to the tibia [28]. Therefore, our results corroborate that the angle between the tibial slope and the tibial axis or the patellar tendon is a major factor for femorotibial stability in CCL deficient joints.

Our findings suggest that there is a biomechanical parallel between dogs and humans with increased posterior tibial slope [27], [29], [30], [31]. In both species, the tibial plateau is not perpendicular to the tibial shaft axis but has a posterior or caudal slope. In humans this slope is 5°–10° and the mean slope in normal dogs is 23°–26° [32]. In humans, the posterior slope causes an anterior directed shear force originating from the tibiofemoral contact force [31]. In our study, as similarly observed in the human knee [31], the cranially directed shear force originating from the caudal slope caused a cranial shift in the tibial resting position. In both dog and human femorotibial joint models changes in anterior or cranial tibial translation are linearly related to a change in the tibial slope [17], [31]. It is conceivable that changes in contact pressure may be similarly related to changes in the tibial slope. Based on this and previous investigations, the dog is a suitable model for the human knee with increased posterior tibial plateau slope.

In our study we found that following transection of the CCL, the peak contact pressure shifted to the caudal aspect of the tibial plateau, leaving areas of the plateau with no contact pressure. These changes are similar to previous investigations on the feline CCL-deficient stifle model that showed that areas of normal contact loading became underloaded, and areas normally subjected to little or no contact pressure became overloaded [21]. Mature cartilage has a location dependent histomorphology that is developed in response to its specific loading history. Variations in cartilage thickness are associated with regional variations in the loading at the knee during walking [13]. Dog stifles have locational variations in cartilage stiffness, which may correspond to the variable loading pattern of the joint [33]. The alterations in load distribution that we observed may be responsible for the early biochemical and morphological articular cartilage changes described in the Pond-Nuki model [34].

Our findings should be carefully interpreted in light of the following limitations. Our study was restricted to an analysis of standing and did not evaluate the dynamic stifle behavior. Static analysis of stance cannot account for potential abnormal kinematics of the joint throughout the entire range of motion. However, previous in vivo kinematic studies have shown that CCL insufficiency results in a consistent pattern of cranial translation and internal rotation of the tibia only during the stance phase of gait [19]. Since both quadriceps and gravitational forces are minimal during swing phase, the primary mechanisms driving cranial tibial displacement are present only during weight-bearing [6]. Another limitation is that our study simulated the acute effects of CCL deficiency. The biomechanics of the CCL-deficient stifle may change over time, due to capsular thickening, osteophyte formation and bone remodeling [35]. Functional and anatomical adaptations may mitigate the acute effects of CCL deficiency over time [36], [37]. It should also be noted that flexion angles differed between intact and CCL deficient stifle in the extended flexion angle. One possible explanation is that we visually aligned the stifle during testing with a goniometer, aiming for approximately 135°: estimation of stifle angle during the experiment were not as accurate during subluxation with CCL deficiency likely because we did not account for the tibial translation and rotation; nevertheless, we were still within 7° of our target, and such minor change was unlikely to have significantly influenced our final results.

The results of this study provide insight into the canine model of CCL deficiency as well as the relationship between stifle alignment and contact mechanics after loss of CCL function. In this model alterations in joint alignment were correlated with shifting of the contact points to infrequently loaded areas of the tibial plateau. Understanding the role of biomechanical alterations responsible for disease progression in experimental models of OA may identify modifiable factors, which will allow surgeons to alter the course of disease.
